# Special Issue: “Bacteriophages and Biofilms”

**DOI:** 10.3390/v13020257

**Published:** 2021-02-08

**Authors:** Zuzanna Drulis-Kawa, Barbara Maciejewska

**Affiliations:** Department of Pathogens Biology and Immunology, Institute of Genetics and Microbiology, University of Wroclaw, 51-148 Wroclaw, Poland

Biofilms are a community of surface-associated microorganisms characterized by the presence of different cell types in terms of physiology and phenotype. These multicellular communities are embedded within a protective matrix of extracellular polymeric substances (EPS) composed essentially of polysaccharides, eDNA, lipids, and proteins. This complex architecture with integrated biomolecules and mineral components serves as a protective layer for hidden bacterial cells, providing high tolerance to antibiotics and biofilm stability [1,2]. Bacterial biofilms are of great medical concern, especially since they are often formed by nosocomial multidrug-resistant pathogens from the ESKAPEE group such as *Staphylococcus aureus*, *S. epidermidis*, *Pseudomonas aeruginosa*, *Klebsiella pneumoniae*, *Enterococcus faecalis* [3,4]. The ability to form biofilms and to modify virulence in response to environmental changes is coordinated by complex bacterial signaling networks including two-component systems (TCS), secondary messengers involved in quorum sensing (QS), and c-di-GMP networks (diguanylate cyclase systems, DGC). Signal cascades dynamically control the transition from the free-living to the sessile mode of growth in response to external environment changes, including viral infection [5].

Bacteriophages, viruses that infect and replicate within bacteria, are perfectly adapted to infect biofilms [6]. Owing to the co-evolution mechanism, phages are actively involved in biofilm formation, in two contradictory ways, as promoting or dispersing agents. Phages can be equipped with matrix-degrading enzymes that allow the effective infection of biofilm embedded cells [7]. In this context, phages are a natural and helpful weapon against microbial biofilms becoming promising alternatives to conventional preparations in therapy, pathogen biocontrol, and food preservation [8]. On the other hand, prophages regulate phage-mediated cell lysis and eDNA release, an important mechanism of stabilizing the biofilm matrix [9].

In this Special Issue, “Bacteriophages and Biofilms”, we were looking for reports and reviews of the most current findings on phage roles in bacterial biofilm formation, maintenance, and degradation. Ten original research articles and one review paper have been published encompassing reports on various aspects regarding phage–host interactions for both planktonic and biofilm-inhabiting cells. The majority of these contributions address the development of novel strategies for preventing or controlling biofilm formation based on natural lytic phages and phage proteins. Some studies dealt with ineffective treatment related to the protective barrier of the biofilm matrix and emergence of phage-resistant variants, showing the overall phage impact on the physiology, architecture, and fitness of biofilm communities.

The reports from Shlezinger et al. [10], Magin et al. [11], and Islam et al. [12] evaluated the potential of bacteriophages in the eradication of planktonic cells and biofilms of vancomycin-resistant *E. faecalis* (VRE), *P. aeruginosa*, and *Samonella*, respectively. These studies showed a high potential of phages in combating pathogenic strains characterized by multidrug resistance and specialized in biofilm formation. Moreover, they shed light on an improved phage–vancomycin combination and overall sensitization of VRE enterococci to antibiotic activity by a possible modification of cell-wall composition [10]. A *Salmonella* phage cocktail turned out to be very effective in bacterial count reduction in milk and poultry meat at room and fridge temperature. It was suggested that phage preparations are promising biological control agents for food preservation as well as food production system maintenance while reducing *Salmonella* biofilm formation on a stainless steel surface [12].

Besides infective phage particles, phage-borne enzymes such as peptidoglycan-degrading lysins (endolysins) are acknowledged as fast and precise antibacterial tools for use against clinically relevant infections and biofilms, especially those caused by Gram-positive representatives [13]. The article provided by Imanishi et al. [14] proves the therapeutic potential of recombinant S25-3 endolysin for staphylococcal impetigo, where the number of intraepidermal *S. aureus* and the size of pustules were reduced in an experimental mouse model. This study shows a regulatory effect of endolysin application on the cutaneous microbiota composition and diversity. There are also more and more reports describing an efficient activity of lysins towards Gram-negatives [15]. The antibiofilm activity of recombinant endolysin LysECD7 originated from *Escherichia* phage ECD7 was tested by Fursov and co-workers against the clinical *K. pneumoniae* strain [16]. The phage-borne lysins turned out to significantly inhibit the formation and degrade mature biofilms in vitro and in a rat model of implant-associated infection [16].

In addition to the antibacterial and antibiofilm potential of phages, three groups also focused on phage–host bacterium interactions and attempted to understand that highly complex issue. Tan and colleagues [17] demonstrated the therapeutic potential of phage cocktails in urinary tract infections caused by multidrug-resistant *K. pneumoniae* ST11. This work paid great attention to the rapid regrowth of phage-resistant variants. Ineffective phage application against staphylococcus biofilms was noted by Melo and co-workers [18], despite the high activity against planktonic cells at different growth stages and lack of inactivation by biofilm matrices. The authors concluded that the *S. epidermidis* biofilm 3D structure and matrix density serve as a shield to protect the embedded bacteria from viral infection. Olszak et al. [19] described the impact of the PA5oct jumbo phage on planktonic cells and biofilms formed by *P. aeruginosa*. The group observed the frequent appearance of resistant clones exhibiting a reduced virulence coupled with sensitization to the innate immune mechanisms. Moreover, the phage resistance was correlated with the phage DNA maintenance (pseudolysogeny) within the bacterial population.

To deepen the knowledge on phage–biofilm interactions, new, advanced and precise methods for monitoring bacterial growth are extremely important. A novel method, based on impedance spectroscopy and quartz tuning forks (QTF), was presented by Gula et al. [20] for *Pseudomonas* biofilm monitoring during phage infection. The authors developed a real-time system able to monitor the physiological changes in the biofilm matrix composition, the regrowth of a phage-resistant population, and the conditions of planktonic cultures to evaluate the activity of antibiofilm compounds.

The last experimental research published in this Special Issue focused on bacterial CRISPR/Cas defense mechanisms against phages and their relationship with bacterial metabolism. Using the *E. coli* model, Yang et al. [21] identified the contribution of the bacterial glycine cleavage system (GCS), serving as a one-carbon provider linked to ATP generation, in *cas3* expression regulation. This study discovered a novel co-regulatory pathway of the CRISPR/Cas3 system encompassing the GCS and cAMP receptor protein (CRP). Therefore, a strict dependence of the *cas3* expression regulation pathway and bacterial defense system on environmental conditions and resource availability was presented.

The therapy objectives of “Bacteriophages and Biofilms” presented in this Special Issue of *Viruses* were summarized by Pinto and colleagues [22] in a review discussing the application of phages in chronic wound treatment. This work focused on the past and ongoing attempts made in vitro, ex vivo, and in vivo using different phage-host systems. The most recent delivery systems developed for the incorporation of phage-based products and ensuring their sustainability were also presented. The effectiveness of phage utilization in the challenging treatment of chronic wound infections in combination with the advances in phage therapy regulation policies should have positive consequences for patients and future health care systems.

## Data Availability

Not applicable.
